# Activity induced delocalization and freezing in self-propelled systems

**DOI:** 10.1038/s41598-018-36824-z

**Published:** 2019-02-04

**Authors:** Lorenzo Caprini, Umberto Marini Bettolo Marconi, Andrea Puglisi

**Affiliations:** 1 Gran Sasso Science Institute (GSSI), Via. F. Crispi 7, 67100 L’Aquila, Italy; 2 Scuola di Scienze e Tecnologie, Università di Camerino - via Madonna delle Carceri, 62032 Camerino, Italy; 3 Istituto dei Sistemi Complessi - CNR and Dipartimento di Fisica, Università di Roma Sapienza, P.le Aldo Moro 2, 00185 Rome, Italy

## Abstract

We study a system of interacting active particles, propelled by colored noises, characterized by an activity time τ, and confined by a single-well anharmonic potential. We assume pair-wise repulsive forces among particles, modelling the steric interactions among microswimmers. This system has been experimentally studied in the case of a dilute suspension of Janus particles confined through acoustic traps. We observe that already in the dilute regime - when inter-particle interactions are negligible - increasing the persistent time, *τ*, pushes the particles away from the potential minimum, until a saturation distance is reached. We compute the phase diagram (activity versus interaction length), showing that the interaction does not suppress this delocalization phenomenon but induces a liquid- or solid-like structure in the densest regions. Interestingly a reentrant behavior is observed: a first increase of *τ* from small values acts as an effective warming, favouring fluidization; at higher values, when the delocalization occurs, a further increase of *τ* induces freezing inside the densest regions. An approximate analytical scheme gives fair predictions for the density profiles in the weakly interacting case. The analysis of non-equilibrium heat fluxes reveals that in the region of largest particle concentration equilibrium is restored in several aspects.

## Introduction

Recently the theorists’ attention has been attracted by the study of so-called self-propelled particles^1–3^ in the context of active matter. Typical experimentally accessible examples come from biological systems: swimming bacteria, such as the E. Coli^4^, unicellular protozoa^5^ and spermatozoa^6^ but also more complex systems such as actin filaments^7^, active nematics^8^, living tissues^9^ or the so-called motor-proteins^10^. Moreover, artificially realized micro-swimmers, such as self-propelled Janus particles^11,12^ and colloidal particles immersed in a bacterial suspension^13^, have been shown to behave as active systems. All these examples show common features both at the level of the single particle trajectory^14^, and at the collective level, which cannot be captured by an equilibrium Brownian motion model. Regardless of their nature, these systems propel themselves in some space direction for a finite time, by employing different mechanisms. Typically, biological systems employ mechanical tools, such as Cilia or Flagella, or complex chemical reactions. Active colloids are typically activated through light^15,16^, which injects energy into the system, or chemically through the decomposition of hydrogen peroxide^17,18^. Independently on their origins, on one hand, an isolated self-propelled particle in absence of any external potentials displays an anomalously long persistent motion, with respect to a Brownian particle. This anomaly is enterely due to the self-propulsion. Of course, at very long times - when velocity correlations have decayed - normal effective diffusion is recovered when active particles are not confined. On the other hand, a suspension of interacting active particles shows interesting collective phenomena such as the so-called motility induced phase separation (MIPS)^19–26^ or dynamical ordering phenomena such as flocking^27^. All these phenomena cannot be explained through an equilibrium approach, i.e. in terms of a Maxwell-Boltzmann distribution. For this reason, a series of simplified models have been recently proposed, in particular, the Run and Tumble model^28–30^ and the Active Brownian Particles (ABP) model^31–33^: the connection between these two modelizations was discussed in^34,35^, showing a good consistency between them, at least in a range of values of the control parameters. Since the two-time correlation of stochastic activity force in the ABP, averaged over the angular degree of freedom, has an exponential shape, the Active Ornstein-Uhlenbeck Particles (AOUP) model was introduced, as the simplest model with such time-correlation behavior^36–40^. Despite its apparent simplicity, many aspects of the active phenomenology were reproduced^37,41^, providing consistency with this model. The possibility in AOUP of obtaining clear theoretical results may lead to new predictions which may trigger future experimental investigations.

With this aim, here we implement numerical simulations^42^ of interacting AOUP particles within a confining single-well potential, reproducing a “delocalization” phenomenon, i.e. the escape of particles far from the potential minimum, recently observed in experiments with Janus particles. In particular, in^43^ the system was dilute enough to make inter-particle interactions negligible. At variance with^44^, our model considers a constant mobility and neglects any kind of hydrodynamic interactions, supposed to be small. Moreover, we do not involve any alignment and consider only pairwise repulsive potentials to model the steric repulsion among the spherical microswimmers. Moreover, in a previous work^45^ a slightly different modelization for the activity is employed which seems to be not consistent with the experimental result of^43^. Our study first demonstrates that delocalization increases with activity and is robust also in the presence of interactions, at least up to a certain effective density. We also reveal a complex interplay between interactions and activity, inducing a freezing phenomenon which is consistent with the one observed with ABP particles in^46,47^. The relative simplicity of the AOUP model allows understanding the physical origin of both delocalization and freezing. In particular, an approximation method, the so-called Unified Colored Noise Approximation (UCNA), well reproduces the density profiles, offering a simple principle for determining the density in the case of non-interacting particles subject to external fields. An interesting observation concerns the role of detailed balance (DB)^48,49^ which is locally satisfied only in regions of space having the highest probabilities of being occupied, while in the remaining regions DB is violated and the local velocity distribution displays strongly non-Gaussian shapes.

## Model and Numerical Results

As mentioned in the Introduction, one of the most popular models describing self-propelled particles is ABP. The microswimmers are approximated as points and the hydrodynamic interactions due to the fluid feedback are neglected. The self-propulsion mechanism is represented by a force of amplitude *v*_0_ and direction ${\hat{{\bf{e}}}}_{i}$. For instance, in two dimensions ${\hat{{\bf{e}}}}_{i}$ is a vector of component (cos *θ*_*i*_, sin *θ*_*i*_), being *θ*_*i*_ the orientational angle of particle *i*. Therefore, the radial component of the activity is assumed to be constant. The ABP dynamics describing a suspension of *N* particles in a two-dimensional system reads:

1 $$\begin{array}{rcl}\gamma {\dot{{\bf{x}}}}_{i} & = & {{\bf{F}}}_{i}+\gamma \sqrt{2{D}_{t}}{{\boldsymbol{\xi }}}_{i}+\gamma {v}_{0}{\hat{{\bf{e}}}}_{i}\\ {\dot{\theta }}_{i} & = & \sqrt{2{D}_{r}}{w}_{i}\end{array}$$

where ***ξ***_*i*_ and *w*_*i*_ are independent white noises (i.e. *δ*-correlated in time and with zero average). *D*_*r*_ is the rotational diffusion coefficient, which states the typical time associated to the activity directional change, *τ*_*r*_ ~ 1/*D*_*r*_. *F*_*i*_ is the total force acting on the particle *i*, which can be decomposed as ${{\bf{F}}}_{i}=-\,{\nabla }_{i}U({{\bf{x}}}_{i})-{\nabla }_{i}{\rm{\Phi }}({{\bf{x}}}_{1},\ldots {{\bf{x}}}_{N})$, i.e. into the force due to the external and to the interaction pairwise potential, respectively. We call *l* and *R*, respectively, the typical length of *U* and Φ, such that ${\rm{\Phi }}={\sum }_{i < j}\,\varphi (|{{\bf{x}}}_{i}-{{\bf{x}}}_{j}|/R)$ and *U* = *U*(**x**/*l*). For the sake of simplicity, *l* is set to one in the numerical study. The parameters *γ* and *D*_*t*_ denote the solvent viscous damping and the bare diffusivity due to thermal fluctuations (i.e. in the absence of forces and activity). Notwithstanding its clarity, deriving further analytical predictions for the ABP model may be difficult even in simple cases. The form of the autocorrelation function, $\langle {\hat{{\bf{e}}}}_{i}(t)\cdot {\hat{{\bf{e}}}}_{j}(t^{\prime} )\rangle$, of the orientational d-dimensional vector ${\hat{{\bf{e}}}}_{i}$ is well known in the theory of rotational diffusion of polar molecules^50^. For generic *d*, averaging over the angular distributions at time *t* and *t*′, we simply obtain ${\langle \langle {\hat{{\bf{e}}}}_{i}(t)\cdot {\hat{{\bf{e}}}}_{j}(t^{\prime} )\rangle \rangle }_{{\rm{\Omega }}}=\exp \,(\,-\,|t-t^{\prime} |{D}_{r}(d-\mathrm{1))}{\delta }_{ij}$, being ${\langle \cdot \rangle }_{{\rm{\Omega }}}$ the average over the angular degrees of freedom. For this reason, as already mentioned in the Introduction, the AOUP model has been introduced as a surrogate able to capture the ABP phenomenology. Indeed, the AOUP model is perhaps the simplest model which exhibits the same two-time correlations matrix as the ABP. In the AOUP one replaces ${v}_{0}{\hat{{\bf{e}}}}_{i}\to {{\bf{u}}}_{i}^{a}$ in Eq. (1), where each component of ${{\bf{u}}}_{i}^{a}$ evolves as an independent Ornstein-Uhlenbeck process. AOUP dynamics reads:

2 $$\begin{array}{rcl}\gamma {\dot{{\bf{x}}}}_{i} & = & {{\bf{F}}}_{i}({{\bf{x}}}_{1},\ldots ,{{\bf{x}}}_{N})+\gamma {{\bf{u}}}_{i}^{a}+\gamma \sqrt{2{D}_{t}}{{\boldsymbol{\xi }}}_{i},\\ \tau {\dot{{\bf{u}}}}_{i}^{a} & = & -{{\bf{u}}}_{i}^{a}+\sqrt{2{D}_{a}}{{\boldsymbol{\eta }}}_{i},\end{array}$$

where ***η***_*i*_ is a d-dimensional noise vector, whose components are *δ*-correlated in time and have unit variance and zero mean. In this approximation the term $\gamma {{\bf{u}}}_{i}^{a}$ represents the self-propulsion mechanism, the internal degree of freedom which converts energy into motion, such that $\langle {{\bf{u}}}_{i}^{a}(t)\cdot {{\bf{u}}}_{j}^{a}(t^{\prime} )\rangle ={D}_{a}/\tau \,\exp \,(\,-\,|t-t^{\prime} |/\tau ){\delta }_{ij}d$. Finally, the non-equilibrium parameters *τ* and *D*_*a*_ are, respectively, the persistence time and the diffusivity due to the activity, which is usually some order of magnitude larger than *D*_*t*_, an approximation often employed also in the ABP model. The identification of the two correlations matrices imposes relations among the coefficients, namely in ${v}_{0}^{2}/d={D}_{a}/\tau$ and *D*_*r*_(*d* − 1) = 1/*τ*. Since the third, fourth, and so on, correlation matrices are in general non-trivial in the ABP, the AOUP model can be considered as its effective Gaussian approximation. Moreover, the unitary constraint of activity is removed meaning that the radial component of the activity has itself a dynamics. As revealed by extensive numerical studies, these approximations seem not to be particularly relevant in order to recover the self-propelled particles phenomenology and for these reasons one could claim the possibility to consider the AOUP as a basic model itself and not simply as an ABP approximation.

We point out that in the potential-free model there are two natural temperatures: the solvent temperature *T*_*b*_ = *γD*_*t*_ and the effective active temperature^51^ *T*_*a*_ = *μ*〈*u*^2^〉 = *μD*_*a*_/*τ* = *γD*_*a*_, where we have defined the effective mass *μ* = *γτ* (see below). We fix the value of *γ* = 1 and inspired to the connection between the AOUP and the ABP model^39^ - we also fix the ratio *D*_*a*_/*τ* = 10 that is the variance of the self-propulsion velocity. This protocol allows us to use a single parameter, *τ*, to tune the relevance of activity in the system. In fact, taking the limit $\tau \to 0$ leads to ${T}_{a}\ll {T}_{b}$, providing a vanishing contribution with respect to the thermal noise. On the contrary, at large values of *τ* one has ${T}_{a}\gg {T}_{b}$: self-propulsion becomes important and the thermal bath can be neglected. We restrict to this second regime, where the system temperature *T*_*a*_ has a limited significance since it represents the temperature of the system only in few specific cases discussed below and in the Supplementary Information (SI). In general, the system is out of equilibrium and many of its statistical properties are hardly comparable to a thermal system.

A well-known result for this model concerns the existence of MIPS in the large activity regime, when $U\equiv 0$ and Φ is given by the sum of pairwise repulsive potentials^41^. In this work, the particles are confined by an external radial potential, *U*(*r*) ∝ *r*^2*n*^ with *r* = |**x**|. We choose *n* > 1 since the case *n* = 1 - when thermal noise and particle-particle interactions are negligible - is trivial even at *τ* > 0, corresponding to a Gibbs density distribution ~${e}^{-U(r)/{T}_{eff}}$ with some temperature *T*_*eff*_ (see discussion after Eq. (3) and SI). If *n* > 1 and *τ* > 0, DB is broken and the steady phase-space distribution is not amenable to a simple representation in terms of *U*(*r*). In the presence of an external potential, a useful dimensionless parameter can be defined:

$${\nu }=\frac{\tau U^{\prime\prime} (l)}{\gamma }.$$

It represents the ratio between the persistence time and the relaxation time due to the external force: *ν* is a relative measure of the activity in our system and determines how far from equilibrium is the system. Indeed, when $\nu \lesssim 1$, the relaxation time of the active force is smaller than the typical time over which a significant change of the microswimmer position, due to the potential, occurs: thus from Eq. (2) we have ${{\bf{u}}}_{i}^{a}\approx \sqrt{2{D}_{a}}{\boldsymbol{\eta }}$. In this case, one recovers an equilibrium-like picture, which can be explained in terms of the effective temperature, *T*_*a*_ = *γD*_*a*_ (SI for more details). When ${\rm{\nu }}\gg 1$, the situation dramatically changes: we have to take into account the dynamics of both degrees of freedom in Eq. (2) and we expect significant departures from an equilibrium-like picture. Note that keeping fixed the strenght of the activity, ${D}_{a}/\tau ={v}_{0}^{2}/d$, *γ* and *U*(*r*), one has that both *T*_*a*_ and *τ* are proportional to *ν*.

### Phase diagram: delocalization and induced freezing

**Figure 1:**
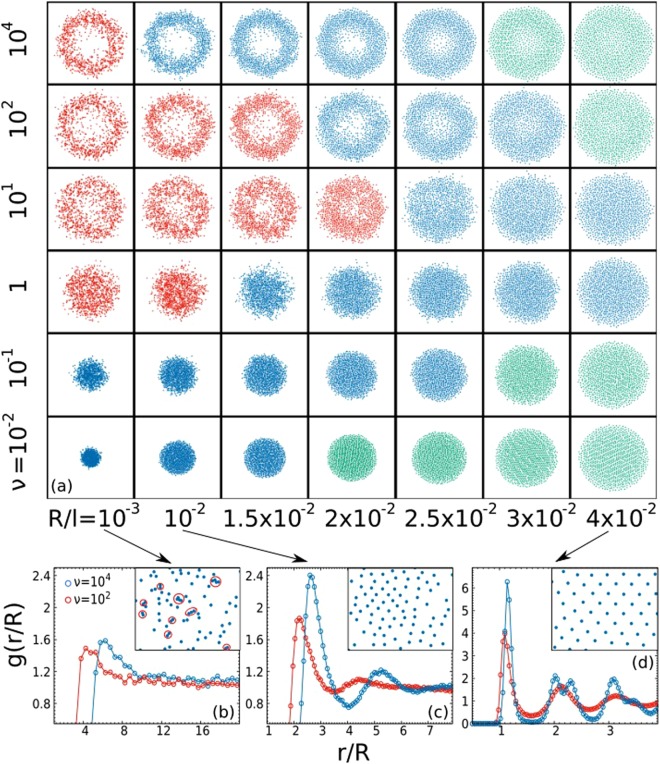
Top: Phase diagram illustrated by simulation snapshots as a function of *R*/*l* and *τ*. Colors indicate the internal density structure (see SI for details): gaseous (red), liquid (blue), solid (green). Bottom: *g*(*r*/*R*) for $R={10}^{-3},{10}^{-2},3\cdot {10}^{-2}$, and for two different values of *τ* = 1, 10^2^, respectively red and blue dots. Each box is realized confining *N* = 10^3^ particles through the interaction potential ${\rm{\Phi }}\sim {\sum }_{i < j}\,{R}^{4}/|{{\bf{x}}}_{ij}{|}^{4}$. Parameters: *n* = 2, *D*_*a*_/*τ* = 10^2^ and *D*_*t*_ = 10^−5^.

In Fig. 1 we display pictorially the phase diagram of a system in 2 dimensions, varying *ν* and the rescaled interaction radius *R*/*l*, (keeping fixed the number of particles and the external potential), which play the role of control parameters. Through *R*/*l* we control the excluded volume of the system, while through *ν* we tune the relevance of the activity ingredient.

Considering the non-interacting regime - Φ = 0 or equivalently *R*/*l* small enough as in the left column of Fig. 1(a) - the equilibrium-like regime for $\nu \lesssim 1$ is consistent with a Brownian-like picture and does not reveal any surprises: particles accumulate around the minimum of the potential, exploring an effective average volume determined just by the interplay between the external potential and the random force. Indeed, the system has effective temperature *T*_*a*_, and no far-from-equilibrium physics is involved. In the non-equilibrium regime, namely $\nu \gtrsim 1$ in Fig. 1(a), the area close to the potential minimum empties and the system shows strong delocalization in favour of a peripheric (annular in 2d) region at an average distance r* from the origin. At large values of *ν*, r* appears to saturate and a further increase of *ν* just produces a dynamical effect, leading to a slowdown of the particles (see SI for details). This phenomenology reproduces the experimental result obtained in^43^ for Janus particles inside an acoustic trap with negligible interactions.

Let us to discuss the interacting case, that is when *R*/*l* is not negligible. The equilibrium-like regime, when $\nu \lesssim 1$ in Fig. 1(a), can be again understood in terms of a Brownian picture. Indeed, the system has temperature *T*_*a*_, regardless of *R*/*l*, and we recover the three equilibrium-like aggregation phases, as expected: a dilute-phase (or gas), where interactions between particles are rare and the volume explored by the particles is only controlled by the random force; a solid-like phase, where the random force is very small compared to the inter-particle interactions and produce only oscillations around the almost-fixed particles positions; and finally; an intermediate liquid-like phase where both these terms are relevant and produce a correlated and complex dynamics. These different internal structures can be roughly identified by the study of the pair correlation function^52^, *g*(*r*), which is estimated by taking into account a region approximately uniform in density, in the densest part of the system (namely the annular region): in the dilute regime *g*(*r*) is flat or “quasi”-flat, in the liquid one *g*(*r*) displays, some peaks before approaching to one and finally in the solid regime these peaks become more pronounced, showing the typical structure of hexagonal lattice (in 2D with radial inter-particles interactions). In all the equilibrium-like aggregation phases the increasing of *ν* produces an expected fluidization phenomenon, which can be easily understood in terms of the effective temperature, *T*_*a*_ ∝ *ν*. In particular, in the liquid-like regime, as shown in the first two left columns of Fig. 1(a), the increase of *ν* enhances the effective volume occupied - when the excluded volume becomes negligible compared to noise-fluctuation -, leading to the transition from the liquid-like to the gas-like structure. In the solid-like regime - last two right columns in Fig. 1(a) -, the interactions are very strong and the effectively occupied volume is determined by the balance between the inter-particle repulsion and the confinement due to the external potential. In this case, the increase of *T*_*a*_ leads only to the fluidization of the internal structure of the system, determining the transition from a solid-like to a liquid-like structure.

Restricted to $\nu \gtrsim 1$, the delocalization phenomenon persists when the interaction radius *R*/*l* increases, as shown in Fig. 1(a). In that case, it is interesting to analyze the internal structure of the system, exploiting analogies and differences with respect to the equilibrium picture. In this regime of *ν*, this analysis leads to the identification of non-equilibrium aggregation phases which resemble the equilibrium scenario but with important differences, which already emerges from the static structure. Indeed, non-equilibrium effects manifest themselves in two ways: 1) in the dilute case - i.e. left column of Fig. 1(a) - a peak at *r* ~ *R* appears in the *g*(*r*), not expected for dilute Brownian particles at the same conditions in terms of density and temperature (Fig. 1(b) and SI for details): this is likely to be similar to that observed in^37^; 2) increasing *R*/*l*, the system displays liquid-like and solid-like structures but with evident shifts in position and intensity with respect to an equilibrium structure with comparable average energy per particle and density, as shown in Fig. 1(c,d) (see SI for details). At large *R*/*l* - but still far from close packing - the system freezes into an almost periodic lattice structure just by increasing *ν* ~ *τ*. This analysis suggests that a purely dynamical quantity, the persistence time, *τ*, can produce a dramatic change in the internal structure of the system. Finally, when the interaction radius *R*/*l* brings the system to an effective close packing, the radial delocalization is completely suppressed and the system comes back to a homogeneous phase with ordered (solid-like) internal structure. In this regime, inter-particle interactions dominate compared to active forces, which are completely negligible.

Summarizing, for all the explored values of *R*/*l*, namely in all the aggregation phases, our numerical study suggests a reentrant behavior of the structural properties of the system induced by *ν*. The first fluidization, explained by the effective temperature approach, is followed by an induced far-from-equilibrium freezing for $\nu \gtrsim 1$, which requires a more subtle analysis to be understood. The discussion, at least regarding the delocalization phenomenon, remains qualitatively valid in three dimensions.

## Theoretical Approach

In order to make analitycal progress, it is common to map Eq. (2) onto a different system, going from the description in the variables $({{\bf{x}}}_{i},{{\bf{u}}}_{i}^{a})$ to $({{\bf{x}}}_{i},{{\bf{v}}}_{i}\equiv {\dot{{\bf{x}}}}_{i})$, i.e. considering the evolution of the coarse-grained velocity of each particle instead of their the activity. When the thermal noise is negligible (i.e. ${D}_{t}\ll {D}_{a}$), deriving with respect to time Eq. (2) and eliminating ${{\bf{u}}}_{i}^{a}$ in favor of **v**_*i*_, leads to^53^ (see also SI):

3a $${\dot{x}}_{i\alpha }={v}_{i\alpha }$$

3b $$\mu \,{\dot{v}}_{i\alpha }=-\,\gamma {{\rm{\Gamma }}}_{ik}^{\alpha \gamma }{v}_{k\gamma }+{F}_{i\alpha }+\gamma \sqrt{2{D}_{a}}{\eta }_{i\alpha },$$

3c $${{\rm{\Gamma }}}_{ik}^{\alpha \gamma }=({\delta }_{ik}+\frac{\tau }{\gamma }\frac{\partial }{\partial {x}_{i\alpha }}\frac{\partial }{\partial {x}_{k\gamma }}({\rm{\Phi }}+U)).$$

where we use Latin and Greek indices for indicating the *N* particle and for the *d* components of the particle coordinates, respectively. We point out that this mathematical passage can be considered only as a change of variables and thus does not involve any approximations. Moreover, *v*_*iα*_ is not the real velocity of such a particle but has to be interpreted just as a coarse-grained velocity, i.e. ${\dot{x}}_{i\alpha }$ by definition, where *x*_*iα*_ is the position of the overdamped dynamics, i.e. such that timescales of molecular interaction and inertia relaxation are filtered out. The original over-damped dynamics of each particle is mapped onto the under-damped dynamics of a particle immersed into a fictitious bath with its effective diffusion coefficient, related to the activity parameters. The non-equilibrium feature of such a dynamics is fully contained in the space-dependent, ${(d\cdot N)}^{2}$ dimensional friction matrix, Γ, which naturally produces a violation of the Fluctuation Dissipation Relation. The dynamics of one particle is coupled to all the degrees of freedom through both the interaction potential and Γ. When particle-particle interactions are negligible, Γ reduces to a *d*-block diagonal matrix, which provides just a coupling among the different components of the dynamics of a single particle. In this case, when $\nu \ll 1$, the Γ matrix reduces to a spatially homogeneous matrix and the system reaches a Gibbs steady state ~exp(−*H*/*T*_*eff*_) with *H* = *μ*|**v**|^2^/2 + *U*(**x**) and *T*_*eff*_ = *T*_*a*_, meaning that *T*_*a*_ can be identified as the effective temperature of the system^54,55^. The peculiarity of the case *n* = 1 emerges in the dilute regime since Γ is constant for all *ν* and *T*_*eff*_ = *T*_*a*_(1 + *ν*)^−1^. In the case *n* > 1 and non-negligible *ν*, only approximations of the stationary pdf^41,56,57^ are known.

The representation of the dynamics given by the Eq. (3) sheds some light on both freezing and delocalization phenomena observed above. The freezing can be understood by the slowing down induced by the increase of Γ, determined by the internal forces among active particles in the large persistence regime. The radial delocalization phenomenon which is observed even in the presence of negligible interactions can be physically understood as follows: the effective damping coefficient, Γ(*x*)/*τ*, is small near the minimum of the potential well and increases as *x* departs from it. Therefore, particles with *x* ≈ 0 can attain large velocities and leave the region, while for *x* large enough they reduce their “effective speed”, *v*, for the combined effects of viscous damping and the external force.

### UCNA approximation

To make this argument quantitative we employ the unified colored noise approximation (UCNA). UCNA was developed first time in^58,59^ in the context of electric fields with a correlated noise, but the methodology has been adapted to interacting active particles systems in^53^. This approximation consists in an effective equilibrium approach which predicts the spatial distribution of the particles in terms of an effective potential, which involves the derivatives of *U* + Φ. The prediction can be derived by dropping the inertial term in Eq. (3) in the limit of vanishing current, or by performing the usual adiabatic elimination in the FP-equation^49^. Its derivation is reviewed in the SI and the final result reads:

4 $$\begin{array}{lll}{p}_{u}({{\bf{x}}}_{1},\ldots ,{{\bf{x}}}_{N}) & \propto  & {e}^{-{H}_{u}({{\bf{x}}}_{1},\ldots ,{{\bf{x}}}_{N})/{T}_{a}},\\ {H}_{u} & = & {\rm{\Phi }}+U+\frac{\tau }{2\gamma }\,\sum _{i\alpha }\,{({F}_{i\alpha })}^{2}-{D}_{a}\gamma \,\mathrm{log}\,|{\rm{\det }}\,{{\rm{\Gamma }}}_{ik}^{\alpha \gamma }|.\end{array}$$

In spite of the fact that the UCNA is derived under the assumption of vanishing currents and thus restores the DB, at least in some regimes it is able to capture many interesting aspects of the observed phenomenology of self-propelled particles.

**Figure 2:**
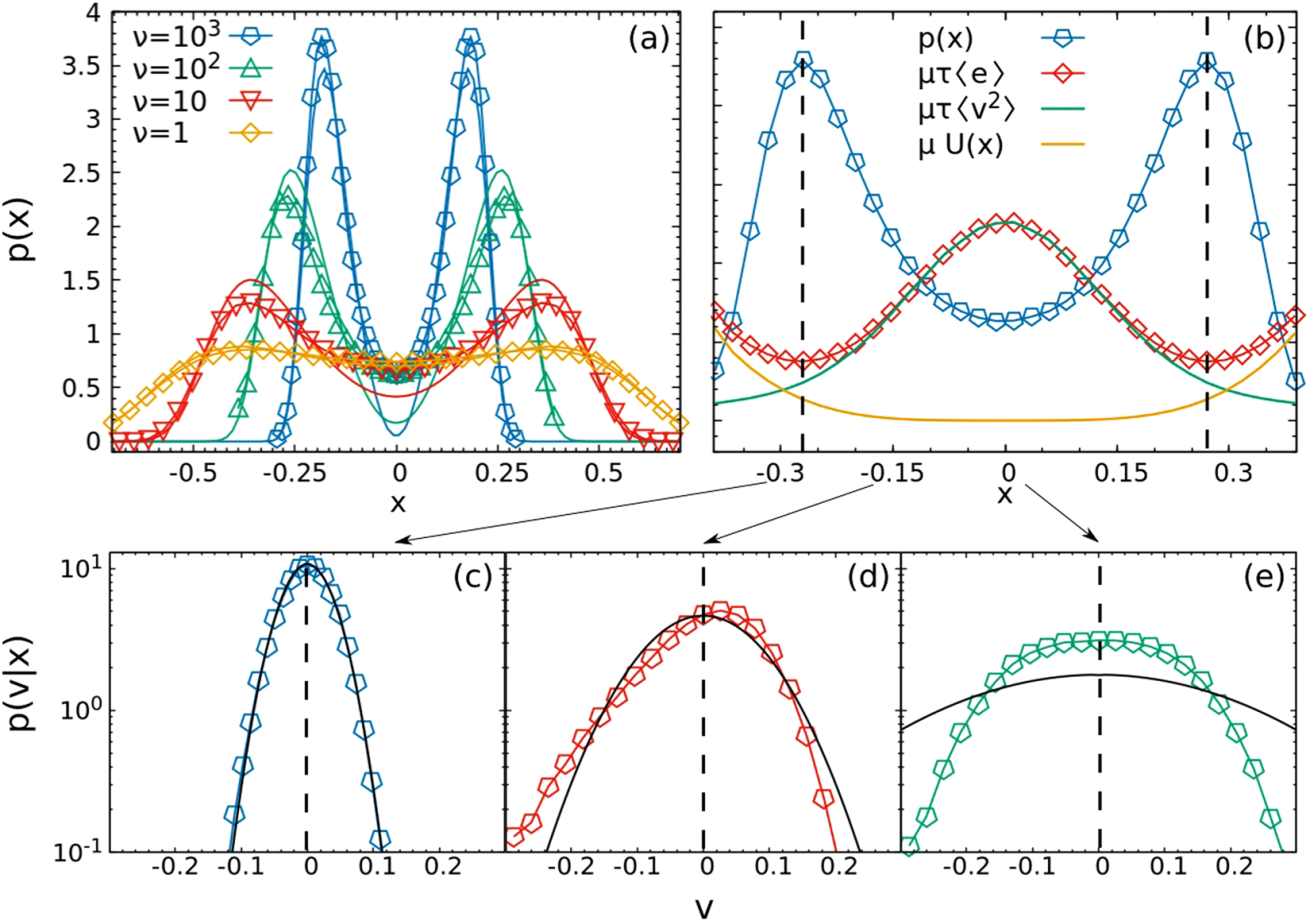
Top Panel: on the left *p*(*x*) computed from data (line + dot) and *p*_*u*_ (line), for different values of *τ*. On the right, for *τ* = 10: *p*(*x*) (blue points), energy 〈*e*〉_*x*_ (red points), 〈*v*^2^〉_*x*_ (green line) and *U*(*x*) (orange line). Two vertical black lines are drawn at *x* = *x*_*m*_, corresponding to the most probable position. Bottom Panel: *p*(*v*|*x*) for three different positions, *x* = −0.3, −0.15, 0, from left to right. The black line is the equilibrium prediction. Parameters: *D*_*a*_ = 1, *τ* = 10, *k* = 10/4, *n* = 2.

In order to assess this approximation, we consider a one-dimensional system of non-interacting particles and show, in Fig. 2(a), the comparison between the numerical probability density in space, *p*(*x*), and *p*_*u*_(*x*). Remarkably, the effective potential *H*_*u*_ takes the shape of a double well which fairly reproduces the numerical simulations. The comparison is optimal when $\tau \ll 1$, and gives fair quantitative information for the location of the density maxima also when $\tau \gg 1$. In particular, *p*_*u*_ correctly predicts the accumulation in some regions, depending on *τ*, but it undererrates the probability of finding a particle in the bottom of the well, for large *τ*. This double-well effective phenomenology may be also related to the results obtained in^60^, explaining why the time-dependent response function of the system shows two different characteristic times for large values of the activity.

### Hydrodynamics

We also consider a hydrodynamic approach, explained in details in ref.^56^, which provides a useful tool to improve the understanding of the observed phenomenon. In particular, let us start from the Fokker Planck (FP) Equation associated to Eq. (3), in 1d in the non-interacting case. Multiplying by a polynomial basis in powers of *v* and integrating out the velocity, we can construct an infinite herarchy of equations, involving the probability density *p*(*x*), the first velocity momentum, 〈*v*〉_*x*_, the second velocity momentum, 〈*v*^2^〉_*x*_, and so on. Here, we have introduced the notation ${\langle \cdot \rangle }_{x}=\int \,dv\cdot p(x,v)/p(x)$, which points out that each observable is an explicit function of the position *x*. The zero-order equation, obtained just by integrating out the velocity in the FP Eq., is equivalent to the mass conservation. The first order equation obtained by multiplying FP by *v* and integrating out the velocity reads:

5 $$\frac{\partial }{\partial t}[p(x){\langle v\rangle }_{x}]+\frac{\partial }{\partial x}[p(x){\langle {v}^{2}\rangle }_{x}]=[\frac{F}{\mu }-\frac{\gamma \,{\rm{\Gamma }}}{\mu }{\langle v\rangle }_{x}]\,p(x).$$

Equation (5) expresses the evolution of the particles momentum, in terms of 〈*v*^2^〉_*x*_ and *p*(*x*). Note that 〈*v*^2^〉_*x*_ is not constant in space, at variance with ordinary underdamped equilibrium dynamics. Iterating this procedure in the polynomial *v*-basis leads to an infinite hyerarchy of equations, which cannot be solved without employing some closure. Since in the stationary state 〈*v*〉_*x*_ = 0, the minima of *p*(*x*) correspond to the minima of the function 〈*e*〉_*x*_ = 〈*v*^2^〉_*x*_ + *U*/*μ*. The slowdown of the particles in regions far from the minima balances the increasing in the potential energy. These results are well verified in Fig. 2(b). Let us notice that the space dependence of 〈*v*^2^〉_*x*_ is determined by the correlation between *x* and *v* and connected with the violation of the detailed balance condition and of the equipartition theorem^41,61^.

### Heat, temperature and local detailed balance

The last observation suggests the existence of non-trivial thermodynamics balances in this system. The analysis of Eq. (3) shows that additional temperature scales exist, which are space-dependent. Their definitions are clear for one particle in one dimension, where Eq. (3b) without external potential takes the form of an equilibrium bath at temperature *θ*(*x*) = *T*_*a*_/Γ(*x*). In the multidimensional case the symmetric matrix Γ can be diagonalised and one obtains a vector of temperatures^62^ (for instance in the radial 2*d* case one has a radial temperature and a tangential temperature). In^56,62^ it was shown that such a temperature satisfies a generalized Clausius relation coupling entropy production and heat exchanged with the bath. In particular, following a stochastic thermodynamics approach^63–67^, the entropy production rate of the medium ${\dot{S}}_{m}$ can be calculated. Despite the recent dispute about ${\dot{S}}_{m}$, the validity of the result was definitively confirmed in^68^. Moreover, ${\dot{S}}_{m}$ and the heat rate density, $\dot{q}(x)$, in 1D, are related through the relation^62^:

6 $${\dot{S}}_{m}=\int \,dx\,p(x)\dot{q}(x)/\theta (x),$$

7 $$\dot{q}(x)=\frac{{D}_{a}\gamma }{\tau \,\theta (x)}\,[\theta (x)-\mu {\langle {v}^{2}\rangle }_{x}],\,\theta (x)=\frac{{D}_{a}\gamma }{{\rm{\Gamma }}}={D}_{a}\gamma \,{(1+\frac{\tau }{\gamma }U^{\prime\prime} (x))}^{-1}.$$

**Figure 3:**
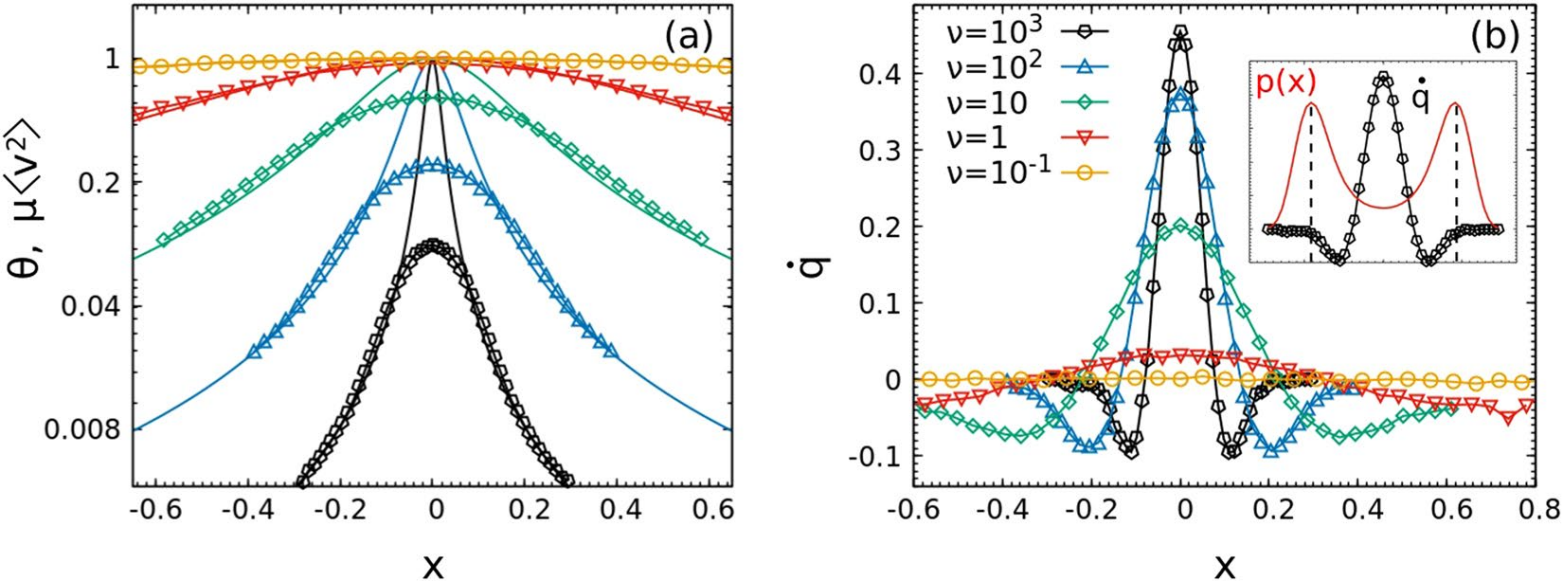
Panel (a): Temperatures *θ*(*x*) (line) and *μ*〈*v*^2^〉_*x*_ (line + dot) in function of *x*. Panel (b): $\dot{q}(x)$, in unit of *D*_*a*_/*τ*, for different values of *τ*. Data are collected through a numerical simulation performed with *N* = 10^4^ independent particles. Parameters: *k* = 10/4, *n* = 2.

Physically speaking, at *x* a local flux of heat is transferred from the system to the active bath if (*μ*〈*v*^2^〉_*x*_ − *θ*(*x*)) is positive while the reverse occurs in the negative case. In the Fig. 3(a), we numerically compare the temperature *θ* and *μ*〈*v*^2^〉_*x*_, showing a clear discrepancy in the central part of the system which increases with *τ*. Interestingly, both temperatures rapidly decrease when moving from the origin to the periphery of the well, making it clear that the annular region where density is high is also very cold. In the proximity of highest density, we have *μ*〈*v*^2^〉_*x*_ ~ *θ*(*x*), meaning that in that region the particles reach an effective thermal equilibrium with the heat bath so that the DB is locally satisfied, although globally it is not. This picture is confirmed by Fig. 3(b) where the local exchange of heat is shown, becoming negligible in the positions corresponding to the density maxima. Therefore, we can identify two symmetric space regions (ER), where the system is almost in equilibrium and others where it is strongly far from it (NER). In order to confirm our intuition, we plot the local conditional probability, *p*(*v*|*x*) = *p*(*x*, *v*)/*p*(*x*), in the bottom graphs of Fig. 2 (Panels (c and d)). The Gaussian prediction at temperature *θ*(*x*) in the ER and a strongly non-Gaussian shape in the NER are confirmed: going towards the origin, *p*(*v*|*x*) becomes an asymmetric function with a skewed tail until the symmetrization is again reached in *x* = 0, where the non Gaussianity is still quite clear. Comparing *p*(*x*) and *p*(*v*|*x*), we note that a particle spends most of its time in the ER, where it accumulates a small amount of heat per unit of time through the coupling with the fictitious bath. When a fluctuation gives it enough energy, it can overcome the effective barrier which separates the two effective symmetric wells, rapidly crossing the NER, and rapidly returning all the heat, absorbed before, to the bath (indeed numerically $\int \,dx\,p(x)\,\dot{q}=0$), in order to come back in the ER.

## Summary and Conclusion

In conclusion, we have reproduced the recent experimental observation of the delocalization phenomenon by means of a simple model of self-propelled particles. We showed that interactions do not suppress the phenomenon (unless close packing is reached) but may induce interesting internal structures which, when self-propulsion is relevant, can be hardly captured by equilibrium modeling and are sensitive to changes of activity time. Interestingly, in the delocalized regime, a local detailed balance condition is verified in the preferred regions. Our conjecture is that this is the reason why the peaks of the density distribution are fairly reproduced by the UCNA approximation in terms of an effective double well potential and an equilibrium-like approach works^69^. Escape times through the effective double well potential could be interesting and improve previous studies^70,71^ where the authors found just a polynomial correction to the Kramers-formula^48^.

## Electronic supplementary material

Supplementary Information
