# Supplementary material for: Activity induced delocalization and freezing in self-propelled systems
Source: Sci Rep. 2019 Feb 4;9:1386. doi: 10.1038/s41598-018-36824-z (PMC6361910; doi:10.1038/s41598-018-36824-z)
Supplement: Supplementary file 1 — Supplementary Information [file 41598_2018_36824_MOESM1_ESM.pdf]

Supplementary Information: Activity induced delocalization and freezing in self-propelled systems

Lorenzo Caprini,[1] Umberto Marini Bettolo Marconi,[2] and Andrea Puglisi[3]
[1]*Gran Sasso Science Institute (GSSI), Via. F. Crispi 7, 67100 L'Aquila, Italy.*
[2]*Scuola di Scienze e Tecnologie, Universitá di Camerino - via Madonna delle Carceri, 62032, Camerino, Italy.*
[3]*Istituto dei Sistemi Complessi - CNR and Dipartimento di Fisica, Università di Roma Sapienza, P.le Aldo Moro 2, 00185, Rome, Italy*
(Dated: October 30, 2018)

In this Supplemental Material, we shall discuss in more detail with respect to the main text (MT) the phenomenology illustrated concerning the radial pair correlation function, $g(r)$, and the concept of effective temperatures of an active system[1–3]. Moreover, we present the derivations of the approximations employed in MT. In particular, in Sec. I we derive Eqs.(3) starting from Eqs.(2) of MT and the Unified Colored Noise Approximation (UCNA), i.e. Eq.(4) of MT. In Sec. II, we discuss the concept of effective temperature applied to the AOUP system[4–7]. In particular, we discuss whether to characterize the system it is appropriate to define the effective temperature, T, through the Gibbs density configurational distribution $\propto e^{-U(r)/T_{eff}}$ or we need alternative definitions, for instance by identifying T with the average kinetic energy of the particles. In Sec. III, we study in detail the $g(r)$ of the system, explaining the computational details and performing an extensive comparison with a Brownian system under the same conditions of density and temperature.

I. DERIVATION OF EQ.(3) & UCNA-APPROXIMATION

In this Section we review for the sake of completeness the derivation of Eqs.(3) of the main text (see refs.[6,11]). Let us start from Eqs.(3) of MT, describing the interacting dynamics of AOUP active particles. Neglecting the thermal noise, these equations read (using Cartesian components and Einstein's summation convention):

$$\dot{x}_{i\alpha} = -\frac{\partial_{i\alpha}\Phi}{\gamma} + u^a_{i\alpha} \tag{1}$$

$$\dot{u}^a_{i\alpha} = -\frac{u^a_{i\alpha}}{\tau} + \frac{\sqrt{2D_a}}{\tau}\eta_{i\alpha} \tag{2}$$

where Ψ is the total potential acting on the system. The Latin index identifies the particles and the Greek index specify the Cartesian component of each vector. Applying the time-derivative to Eq.(1) and defining the coarse-grained velocity:

$$v_{i\alpha} = \dot{x}_{i\alpha}, \tag{3}$$

we obtain:

$$\ddot{x}_{i\alpha} = -\frac{v_{j\beta}}{\gamma}\partial_{j\beta}\partial_{i\alpha}\Phi + \dot{u}^a_{i\alpha} = -\frac{v_{j\beta}}{\gamma}\partial_{j\beta}\partial_{i\alpha}\Phi - \frac{u^a_{i\alpha}}{\tau} + \frac{\sqrt{2D_a}}{\tau}\eta_{i\alpha} \tag{4}$$

where in the last equality we have used Eq.(2) to eliminate $\dot{u}^a_{i\alpha}$. Now, using Eq.(1) and (3) to eliminate $u^a_{i\alpha}$ we obtain:

$$\dot{v}_{i\alpha} = -\frac{v_{j\beta}}{\gamma}\partial_{j\beta}\partial_{i\alpha}\Phi - \frac{\partial_{i\alpha}\Psi}{\gamma\tau} - \frac{v_{i\alpha}}{\tau} + \frac{\sqrt{2D_a}}{\tau}\eta_{i\alpha} = -\frac{\partial_{i\alpha}\Psi}{\gamma\tau} - \frac{1}{\tau}\left[\delta_{ij}\delta_{\alpha\beta} + \frac{\tau}{\gamma}\partial_{j\beta}\partial_{i\alpha}\Phi\right]v_{j\beta} + \frac{\sqrt{2D_a}}{\tau}\eta_{i\alpha} \tag{5}$$

which is Eqs.(3) of MT, being $\Gamma_{ij\alpha\beta} = \delta_{ij}\delta_{\alpha\beta} + \frac{\tau}{\gamma}\partial_{j\beta}\partial_{i\alpha}\Phi$.

We can derive the Unified colored noise approximation (UCNA) by taking the over-damped limit, $\dot{v}_{i\alpha} \approx 0$, in Eq.(5). This procedure leads to a relation between v and x:

$$\dot{x}_{i\alpha} = v_{i\alpha} = \Gamma^{-1}_{ij\alpha\beta}\left[-\frac{\partial_{j\beta}\Psi}{\gamma} + \sqrt{2D_a}\eta_{j\beta}\right] \tag{6}$$

ruling the UCNA dynamics. Eq.(6) involves a multiplicative noise and does not satisfy the fluctuation-dissipation theorem. The associated Fokker-Planck equation associated to Eq.(6) - using the Stratonovich integration - , for the density $p(\{x\}, t)$, reads:

$$\partial_t p = -\partial_{i\alpha}J_{i\alpha}, \qquad J_{i\alpha} = -\Gamma^{-1}_{i\alpha k\beta}\left[\partial_{k\beta}\Psi\, p + D_a\partial_{k\beta}\left(\Gamma^{-1}_{i\alpha j\beta}\, p\right)\right] \tag{7}$$

Looking for a stationary solution of Eq. (7) under the condition of vanishing current we find the following equation:

$$-p\,\frac{\partial_{k\alpha}\Psi}{D_a\gamma}-p\;\partial_{j\beta}\Gamma^{-1}_{j\beta i\alpha}=\Gamma_{j\beta i\alpha}[\partial_{j\beta}p]. \tag{8}$$

After some algebra we obtain:

$$-p\,\frac{\tau}{D_a\gamma}\Gamma_{i\alpha j\beta}\partial_{j\beta}\Psi+p\;\Gamma^{-1}_{j\beta k\delta}\partial_{j\beta}\Gamma_{k\delta i\alpha}=\partial_{i\alpha}p \tag{9}$$

Moreover, by using the identity, $\partial_{j\beta}\Gamma_{i\alpha k\gamma}=\partial_{k\gamma}\Gamma_{i\alpha j\beta}$ - since Γ involves only the second derivatives of Ψ - and the Jacobi's formula:

$$\frac{1}{\det\Gamma}\partial_y\det\Gamma=Tr\left(\Gamma^{-1}\partial_y\Gamma\right) \tag{10}$$

we obtain:

$$\Gamma^{-1}_{i\alpha j\beta}\partial_{i\alpha}\Gamma_{j\beta k\delta}=\frac{1}{\det\Gamma}\partial_{k\delta}\det\Gamma \tag{11}$$

Using this result in Eq.(9):

$$-D_a\gamma\left(\partial_{i\alpha}p-p\;\partial_{i\alpha}\log\det\Gamma\right)=p\;\Gamma_{i\alpha j\beta}\partial_{k\beta}\Psi \tag{12}$$

Solving this set of partial first-order differential Eqs. we find the UCNA probability distribution:

$$\begin{aligned}&p\propto\exp\left(-\frac{\mathcal{H}}{D_a\gamma}\right),\\&\mathcal{H}=\Psi+\frac{\tau}{2\gamma}\sum_{k\beta}\left(\partial_{k\beta}\Psi\right)^2-D_a\gamma\log\det\Gamma.\end{aligned} \tag{13}$$

Considering just the one-dimensional case in the non-interacting case, we find the pdf $p(x)$ shown in Fig.2 of MT

The possibility of neglecting $\dot{v}=0$ in Eq.(5), i.e. taking the overdamped limit, is equivalent to assume the Gaussianity of the conditional probability, $p(v|x)$, with a kinetic "effective temperature" which satisfies the Einstein relation. The form of such a $p(v|x)$ shows that different components of the velocity are not independent. In fact, the approximate probability distribution reads:

$$p(\{x\},\{v\})=p(\{v\}|\{x\})p_u(\{x\})\propto e^{-v_{i\alpha}\Gamma_{ij\alpha\beta}v_{j\beta}}p_u. \tag{14}$$

We point out that Eq.(14) is not the solution of the FP-equation associated to Eq.(5), but can be just considered as a useful approximation. Despite its apparent simplicity, it involves many-body interactions, which cannot be easily evaluated and for this reason, up to now, the UCNA was not particularly practical in understanding collective phenomena.

II. A KINETIC TEMPERATURE FOR THE ACTIVE SYSTEM

In the presence of an external potential, it is not clear which should be the temperature of an assembly of active particles[8]. Recently, some approximations were developed with the aim of describing by an effective potential the particles interactions[9–12]. These approximations seem to work in spite of the fact that these systems are clearly far from equilibrium[13,14].

As discussed in MT, the potential-free system displays two temperatures: $T_b=\gamma D_t$ determined by the solvent, and $T_a=\gamma D_a$, (the so called active temperature) related to the self-propulsion force, γu. Since we fix the variance of u, i.e. the ratio D_a/τ, the possibility of neglecting T_b with respect to T_a depends on the value of τ. On one hand, for τ small enough $T_a\ll T_b$, one encounters a non-interesting regime where the activity plays a negligible role and the system behaves as if it were subject to Brownian dynamics, at temperature T_b. On the other hand, the more interesting regime studied in the main text occurs when τ is large enough, i.e. $T_a\gg T_b$, so that we can effectively neglect the solvent temperature. The presence of a non uniform external force leads to a new effect: it determines a non-trivial correlation between the position, x, of the particle and its self-propulsion, γu, which eventually leads to the violation of the equipartition theorem[14,15], breaking the DB[16]. In this case, the identification of T_a with an

effective temperature[8,17] is not trivial and depending on the choice of the parameters in general not true. In MT, we exploit the importance of ν - i.e. the ratio between the typical time τ associated with the active force and the one associated with the potential, $\gamma/U''(l)$. The dimensionless parameter ν is recognized as the relevant parameter determining if the system is close to a global equilibrium. In particular, if $\nu \ll 1$ but $T_a \gg T_b$ we can perform the overdamped limit of the Eq.1(a) of MT, approximating u as a Brownian process. This operation provides a simplified overdamped dynamics for the particle position, meaning that the system reaches the equilibrium, evolving with an effective Brownian dynamics with diffusion coefficient, D_a:

$$\dot{x} = -\frac{U'(x)}{\gamma} + \sqrt{2D_a}\eta \qquad \longrightarrow \qquad p(x) \propto \exp\left[-\frac{U(x)}{\gamma D_a}\right]. \tag{15}$$

In this regime, $T_a = \gamma D_a$ has, trivially, the role of the effective temperature of the system. For $\nu \sim O(1)$, this is no longer true, since the system is not in the overdamped regime. Moreover, we can directly check this claim by evaluating a simple solvable case: the harmonic potential in one dimension. Indeed, by setting $U(x) = kx^2/2$, Eqs.(2) of MT can be solved[4,7], providing an analytical expression for the steady state probability $p(x,u)$ for all values of ν:

$$p(x,u) \propto \exp\left[-\frac{k}{\gamma}\frac{\Gamma}{D_a}\frac{x^2}{2}\right] \exp\left[-\frac{\tau}{D_a}\frac{\Gamma}{2}\left(u - \frac{k}{\gamma}x\right)^2\right], \qquad \Gamma = 1 + \nu \tag{16}$$

As we can see, T_a does not coincide with the effective temperature of the system when ν is not negligible, a result which is in general true for a generic potential, except for some special cases[4].

The UCNA equilibrium-like approach, employed in MT and reviewed in Sec.I, provides a prediction for the equilibrium temperature $\theta(x)$ in the non-interacting one dimensional system: $\theta(x) = \gamma D_a/\Gamma(x)$, being $\Gamma(x) = 1 + U''(x)\tau/\gamma$. In particular, we find numerically that UCNA does not hold globally in space but only in the so-called equilibrium regions (ER), which correspond to the regions where the particles spend most of their life, as shown in MT. In the ER the stationary probability distribution, $p(x,v)$, is a Gaussian with respect to v:

$$p(x,v) \propto e^{-H_u/D_a\gamma} e^{-\mu v^2/2\theta(x)}, \qquad H_u = U(x) + \frac{\tau}{2\gamma}\left[\frac{\partial}{\partial x}U(x)\right]^2 - \gamma D_a \log[\Gamma(x)], \tag{17}$$

adapting Eq.(14) to the one-dimensional non-interacting case. Therefore, $\theta(x)$ can be interpreted as a space dependent kinetic temperature. For small activity, θ is almost equivalent to T_a, but this is no longer true at large activity since the space-dependence plays an important role.

III. SPATIAL STRUCTURE AND THERMAL EQUIVALENTS OF THE ACTIVE SYSTEM

In Fig.1 of the main text, we have reported the important changes of $g(r)$ when R/l and ν are varied. Here, we discuss the possibility of interpreting these changes in terms of some effective temperature. Let us see what happens to $\theta(x)$ which, according to Section 1, can be interpreted as an effective local kinetic temperature.

As discussed in Section 1, θ scales as $D_a\gamma/\tau$ when $\nu \gg 1$, meaning that the increasing of ν, leaves unchanged this effective temperature of the system since the ratio D_a/τ is fixed. In the bottom panel of Fig.(1) of the main text, we display $g(r)$ for different interaction lengths, R/l, and for two values of $\nu = 10^2, 10^4$. In all cases, a freezing phenomenon seems to occur with the increase of ν, since the peaks of $g(r)$ become more pronounced. We point out that, on one hand, these measures were performed by monitoring the effective density of the system in the more crowded regions: ρ remains nearly constant in such a way that its variation cannot be considered the cause of the structural changes appearing in $g(r)$. Also θ roughly does not change, meaning that such structural changes are not driven by a variation of θ.

We try an alternative approach and look whether an equivalent Brownian system exists - at the same density and appropriate temperature - displaying the same $g(r)$. To answer these questions boils down to establish whether there exists or not a mapping between the active system and a fictitious over damped passive system.

For an equilibrium system of passive interacting Brownian particles ($D_a = 0$), with diffusion coefficient D_t, the Einstein relation holds and we can identify γD_t as the temperature of the system, T_b. At fixed area fraction, a variation of T_b produces a change in the structure of the system, which can be analyzed by the pair correlation function, $g(r)$[19]. For T_b large enough the $g(r)$ is flat, meaning that there are not preferential distances among particles, a situation which can be roughly identified as a gas-like phase. Particles move around the accessible volume and the interactions are rare and binary-like. The decreasing of T_b, produces some peaks in the $g(r)$, before approaching to one. These peaks establish the typical distances among particles, a regime identified as liquid-like, since particles move around

the available volume and particles positions are strongly correlated. A further decreasing of T_b leads to a freezing pattern, where particles just fluctuate around their fixed equilibrium positions, which are hexagonally distributed in two dimensions. The $g(r)$ peaks become higher and thin, approaching to δ-Dirac function (ideally at $T_b = 0$), a phase which has strong analogies with a solid. The same qualitative picture, gas $\to$ liquid $\to$ solid, is, roughly, produced by the growth of the packing fraction of the system, $\phi \approx \rho_0 R^2$ (in two dimensions), being ρ_0 the numerical density and R the interaction length of the repulsive pairwise potential. Let's remark that the identification of the microscopic structures with the macroscopic phases (eventually with phase transitions) makes sense just if we consider the infinite volume limit. If we apply an external potential, particles can explore just an effective volume, depending both on the inter-particle interactions and on the potential itself. Therefore, fixing the number of particles to $\sim 10^2 - 10^4$ (typical numbers of a simulation), means to study a system with few degrees of freedom, whose importance, nowadays, is well known. With these motivations, studying the internal structure of such a system, for instance through the $g(r)$, makes sense and could be useful in order to understand the role of the interactions.

In Fig. 1 of the SM we compare the $g(r)$ of active systems with that of many possible passive systems having the same interactions and density (for details see Sec. III A), varying the diffusion coefficient. From this analysis it emerges that in the gas-like regime (Fig. 1 panel (d)), when R is very small, $g(r)$ displays a first peak at $r \approx R$, which does not have a Brownian counterpart. Indeed, the passive $g(r)$ profiles are flat also for very low temperatures, which is not a surprise since the interactions are rare. We interpret the active peak as a consequence of the slow-down of the particles in the presence of a convex interaction, as $\sim 1/|\mathbf{x}|^b$ with $b > 0$, which increases the probability that two particles are close to each other. This is the leading mechanism on which the MIPS phenomenon is based.

In the top panel of Fig. 1, we perform the same analysis with an interaction R larger than the previous case, such that a liquid-like structure is produced as the pronounced peaks reveal. For $\nu \geq 1$, as shown in panels (b) and (c), again it is not possible to determine a value of the diffusion coefficient in such a way that we can reproduce the shape of $g(r)$ in the active case: indeed, the active peak is always shifted towards smaller values of r. Such an effect clearly disappears when $\nu \ll 1$ since in the last case an active system is equivalent to a passive overdamped one with effective temperature T_a (Fig 1 panel (a)).

Finally, a further increasing of R, leads to a completely different scenario also for $\nu \sim O(1)$. Although the effective temperature of the system does not trivially scale with ν, the comparison with a Brownian simulation shows that we can find a numerical temperature value, T_r, through which we can reproduce the active $g(r)$ shape, as illustrated in Fig. 1 panel (e).

We may conclude that when the packing fraction is large enough the microscopic structure, represented by $g(r)$, is the same as the one of an equivalent Brownian system with an effective temperature, T_r. Nevertheless, there is no way to reproduce the active $g(r)$ in the gas-like and liquid-like regime induced by the activity, with an equivalent Brownian simulation under the same condition: these structural changes are entirely due to the activity and are genuine non-equilibrium effects.

A. Details about the computations

In the active case, we have computed numerically the $g(r)$ function by using the following procedure:

1. We chose a square inside the dense region of a configuration: a disk or a circular crown (depending if the radial delocalization occurs or not). This square is chosen not too big, in such a way we may neglect the boundary of such a region.

2. We compute numerically the $g(r)$ in this region, using the standard formula[19]:

$$g(r) = \frac{V}{N} \left\langle \frac{1}{N} \sum_{i \neq j} \delta[r - (r_i - r_j)] \right\rangle$$

, where r_i is the position of a target particle and $\langle \cdot \rangle$ denotes both the average over all the particles inside the region and a time average. The normalization is estimated by numerically computing the number of particles inside the square for each configuration.

3. Consistency check: in order to check the result, we perform the same analysis for different (but dense) regions, verifying that there are no big changes.

In the following, we describe the protocol adopted in order to compare an AOUP system with a Brownian one. In particular, we are interested in performing a Brownian simulation under the same conditions of an active system, i.e. same packing fraction and number of particles:

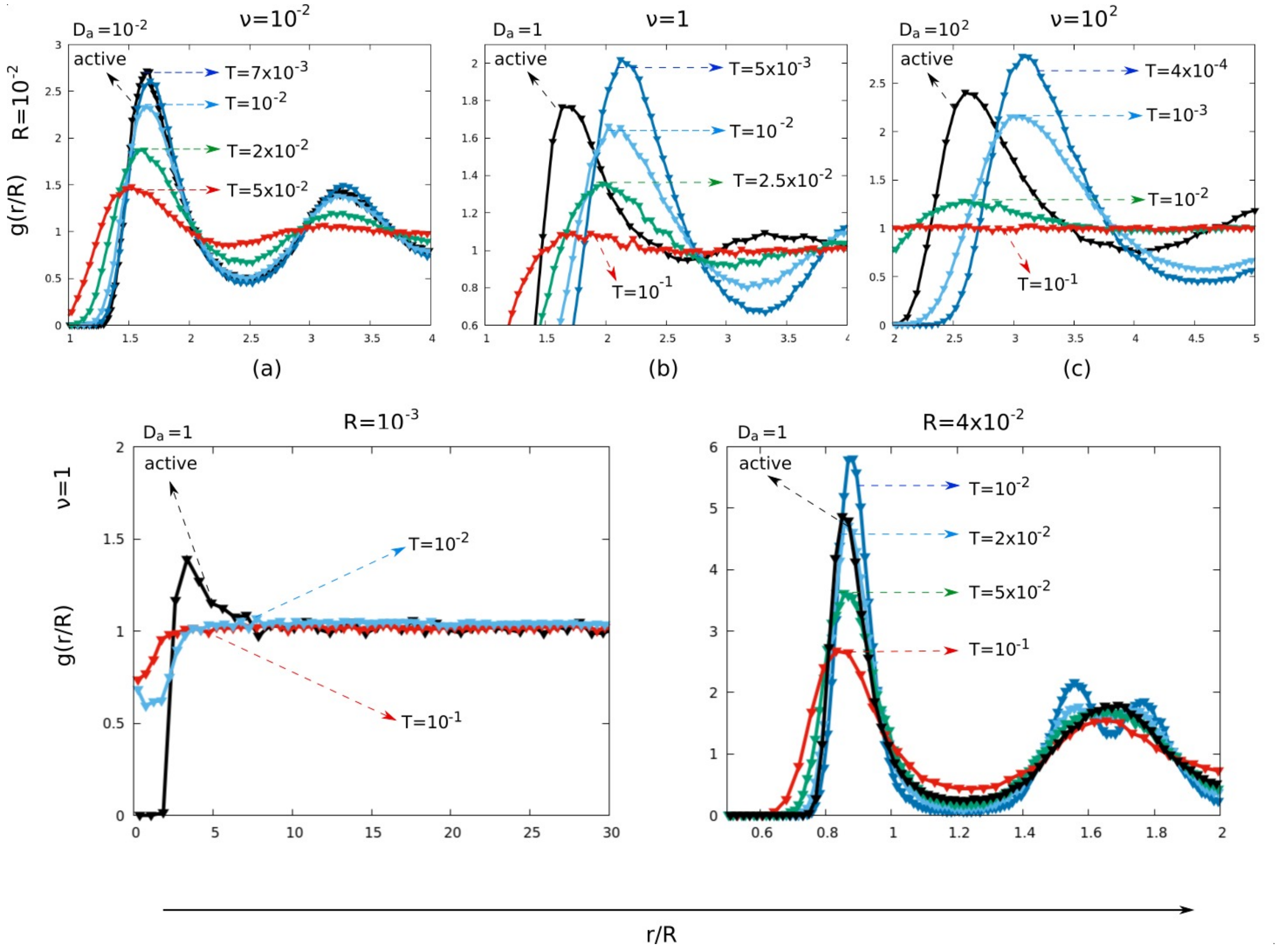

FIG. 1: $g(r)$ for an active system (black line) compared with the equivalent Brownian systems at different temperatures (colored line as shown in each graph). Top Panel: $R = 10^{-2}$, graph(a),(b) and (c), respectively, at $\nu = 10^{-2}, 1, 10^2$. Bottom panels: $\nu = 1$. Graph(d) at $R = 10^{-3}$ and graph(e) at $R = 4 \cdot 10^{-2}$. Other parameters: $k = 10^2/4$, $D_a/\tau = 10^2$, $b = 4$.

1. We compute the $g(r)$ in the active case, with the procedure described above, by selecting a square space region, of area A_r, and computing the average number of particles, N_r, in that region.

2. We compute the $g(r)$, for the following system: N_r overdamped Brownian interacting particles in a square region of area, A_r, under the action of the confining potential. In this way, the numerical density of the equivalent Brownian system is the same as the AOUP. Considering the same interactions we have a Brownian system with the same packing fraction.

3. We compute the $g(r)$ of the Brownian system for different values of the diffusion coefficient, checking if there is some temperature value which reproduces the active $g(r)$.

---

[1] Bechinger, C. et al. Active particles in complex and crowded environments. *Reviews of Modern Physics*, **88** (4):045006, 2016.

[2] Ramaswamy, S. The mechanics and statistics of active matter. *Annual Review of Condensed Matter Physics*, **1**,323, 2010.

[3] Marchetti, M.C. et al. Hydrodynamics of soft active matter. *Reviews of Modern Physics*, **85** (3):1143, 2013.

[4] Szamel, G. Self-propelled particle in an external potential: Existence of an effective temperature. *Phys. Rev. E*, **90** (1):012111, 2014.

[5] Maggi, C., Marini Bettolo Marconi, U., Gnan, N. & Di Leonardo, R. Multidimensional stationary probability distribution for interacting active particles. *Sci. Rep.*, **5**, 2015.
[6] Marini Bettolo Marconi, U., Gnan, N., Paoluzzi, M., Maggi, C. & Di Leonardo, R. Velocity distribution in active particles systems. *Sci. Rep.*, **6** (33):23297 EP, 2016.
[7] Das, S., Gompper G. & Winkler, R. G. newblock Confined active Brownian particles: theoretical description of propulsion-induced accumulation. *New J. Phys.* **20**, 015001, 2018.
[8] Puglisi, A., Sarracino, A. & Vulpiani, A. Temperature in and out of equilibrium: A review of concepts, tools and attempts. *Phys. Rep.*, **709-710**, 2017
[9] H'walisz, L., Jung, P., Hänggi, P., Talkner, P. & Schimansky-Geier, L. Colored noise driven systems with inertia. *Zeitschrift für Physik B Cond. Mat.*, **77** (3):471–483, 1989.
[10] Hanggi, P. & Jung, P. Colored noise in dynamical systems. *Advances in Chemical Physics*, **89**: 239–326, 1995.
[11] Marini Bettolo Marconi, U. & Maggi, C. Towards a statistical mechanical theory of active fluids. *Soft matter*, **11** (45):8768–8781, 2015.
[12] Wittmann, R. et al. Effective equilibrium states in the colored-noise model for active matter II. A unified framework for phase equilibria, structure and mechanical properties. *J. Stat.* **2017** (11), 113207, 2017.
[13] Fodor, È. & Marchetti, M. C. newblock The statistical physics of active matter: From self-catalytic colloids to living cells *Physica A: Statistical Mechanics and its Applications*, **116** (3), 2018.
[14] Fodor, É. et al. How far from equilibrium is active matter? *Phys. Rev. Lett.*, **117** (3):038103, 2016.
[15] Maggi, C., Paoluzzi, M., Angelani, L. & Di Leonardo, R. Memory-less response and violation of the fluctuation-dissipation theorem in colloids suspended in an active bath. *Sci. Rep.* **7** (1), 2017.
[16] Marini Bettolo Marconi, U., Puglisi, A. & Maggi, C. Heat, temperature and Clausius inequality in a model for active Brownian particles. *Sci. Rep.*, **7**, 2017.
[17] Cugliandolo, L. F. The effective temperature *J. of Phys. A*, **44** (48), 2011.
[18] Caprini, L., Marini Bettolo Marconi, U. & Vulpiani, A. Linear response and correlation of a self-propelled particle in the presence of external fields *J. Stat.* **2018**, (3), 033203, 2018.
[19] Lowen, H. Melting, freezing and colloidal suspensions. *Phys. Rep.* **237**, 249-324 1994.
